# Green-Extension Design—A New Strategy to Reduce the Environmental Pressure from the Existing Consumer Electronics

**DOI:** 10.3390/ijerph18189596

**Published:** 2021-09-12

**Authors:** Siliang Yi, Chih-Fu Wu

**Affiliations:** 1 The Graduate Institute of Design Science, Tatung University, Taipei 104, Taiwan; 2 Department of Industry Design, Tatung University, Taipei 104, Taiwan; wcf@ttu.edu.tw

**Keywords:** Green-Extension Design, environment, product life, competitiveness component

## Abstract

Existing electronic devices will quickly become e-waste when encountering technological iterations, which results in serious environmental and public health problems. Previous circular economy research has mainly focused on the development of new products with long life or recycling discarded products. This study firstly proposes the Green-Extension Design (GED) strategy for developing adaptable accessories that provide existing products with the ability to continue to work in a different context. Competitiveness was selected to evaluate the performance of GED, and three competitiveness components were derived through principal component analysis (PCA). Moreover, AHP (Analytic Hierarchy Process) was applied to define the weights of the three competitiveness components, and a GED model was established on the basis of production function. Furthermore, the calculation method for each competitiveness component was defined. The GED strategy is aimed at extending the life of existing products, as well as reducing resource waste and environmental pollution. The GED model based on competitiveness components can enable enterprises to design products of high competitiveness and obtain market share as a result.

## 1. Introduction

Life and work have developed toward a higher degree of automation with the advancement of technology, which has increased the use of electrical and electronic equipment; therefore, electrical and electronic products have become common in the daily lives of ordinary consumers.

At the same time, the pressure of e-waste on the environment is increasing. It was reported by Baldé et al. [1] that approximately 49.8 million tons of e-waste has been attained worldwide, estimated to increase to 51.8 million tons by 2020. Furthermore, the development of more advanced, faster, and more reliable technologies has led to a shortening of product life cycles, prompting consumers to buy newer products and discard old products. All these developments in turn have led to an exponential increase in e-waste generation. For example, the average lifespan of a new computer was reduced from 4.5 years in 1992 to about 2 years in 2005, and it is still declining [2]. The estimated annual growth rate of e-waste flow is 3–5% [3], which is three times faster than other waste streams [4].

**Figure 1:**
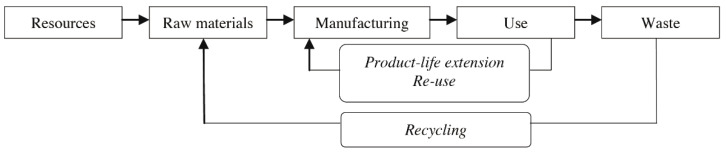
Different types of loops within a closed-loop system [11].

The circular economy is considered a promising way to help reduce our global sustainability pressures [5], which has gained momentum among both scholars and practitioners [6]. A closed cycle of flows can only be sustained as long as its external energy supply lasts. A logical consequence of striving to create closed-loop systems is that there are only two possible long-term fates for waste materials: recycling and reuse or dissipative loss of resources, such as for lubricants or detergents [7]. When comparing linear and cyclical approaches for the development of products and systems, Braungart et al. [8] distinguished between “cradle-to-grave” flows of materials and cyclical, “cradle-to-cradle” flows. In addition, Stahel [9] distinguished between two fundamentally different types of loops within a closed-loop system: (1) reuse of goods and (2) recycling of materials, as shown in Figure 1. The result of goods reuse is a reduction in the flow of materials from production to recycling [10].

**Figure 2:**
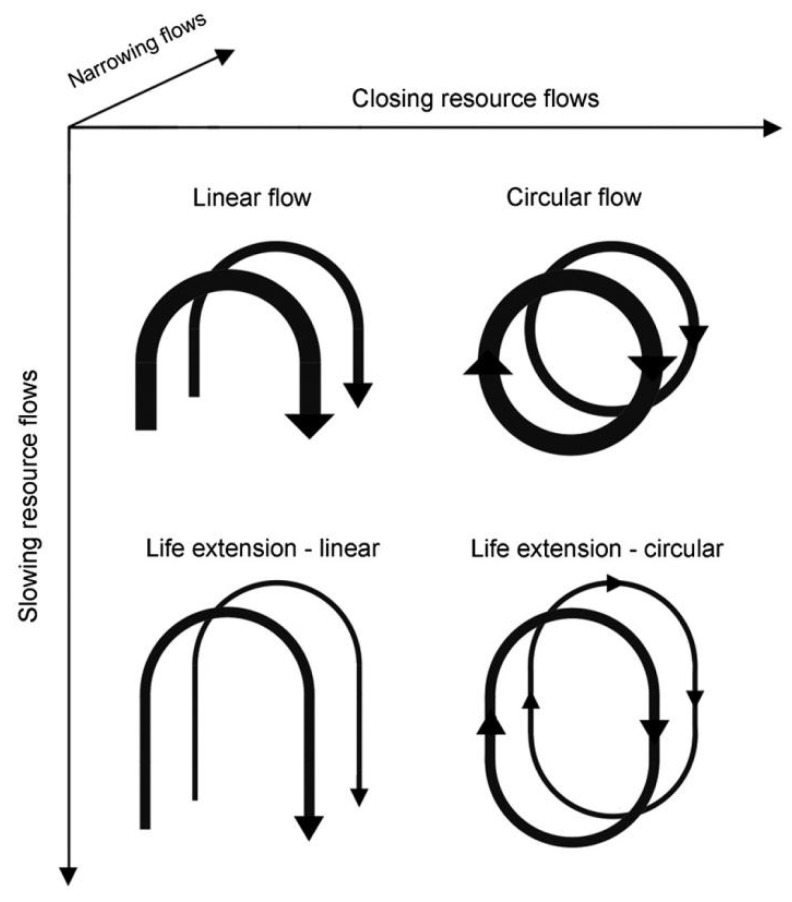
Categorization of linear and circular approaches for reducing resource use. From Nancy et al. [12].

On the basis of the work of Stahel and Braungart et al., Nancy [12] proposed two design approaches to extend product life and slow down resource circulation: (1) slowing resource loops and (2) closing resource loops. Slowing resource loops denote that, through the design of long-life goods and product-life extension (i.e., service loops to extend a product’s life, for instance, through repair and remanufacturing), the utilization period of products is extended and/or intensified, resulting in a slowdown of the flow of resources. Closing resource loops denote that, through recycling, the loop between post-use and production is closed, resulting in a circular flow of resources. These two approaches are distinct from a third approach toward reducing resource flows: (3) resource efficiency or narrowing resource flows, aimed at using fewer resources per product. These three approaches are illustrated in Figure 2.

Several strategies related to extending product life have been explored by previous studies aiming at slowing resource loops. One stream involves designing long-life products. “Designing for reliability” refers to designing products for a high likelihood that they will operate throughout a specified period without experiencing a chargeable failure, when maintained in accordance with the manufacturer’s instructions [13]. “Designing for attachment and trust” refers to the creation of products that will be loved, liked, or trusted longer. This is also referred to as “designing for emotional durability”, a situation where “users and products flourish within long-lasting empathic partnerships” [14]. Another stream involves designing for maintenance and repair. Maintenance is the performance of inspection and/or servicing tasks (technical, administrative, and managerial) [15] to retain the functional capabilities of a product. Repair is related to restoring a product to a sound/good condition after decay or damage [16]. The third stream involves designing products to allow for future expansion and modification. Upgradability is defined as the ability of a product to continue being useful under changing conditions by improving the quality, value, and effectiveness or performance. For example, designing for standardization and compatibility can create products with parts or interfaces that also fit other products.

**Figure 3:**
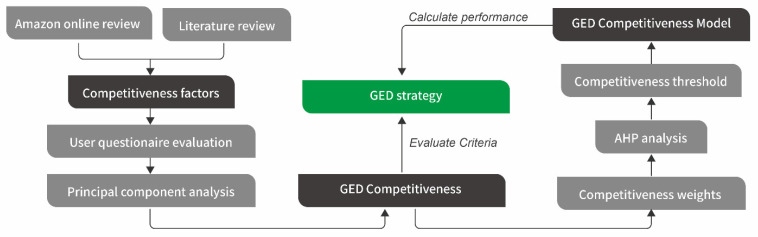
The framework of the GED strategy and its evaluation.

Extending the utilization period of products can be a highly effective strategy for reducing the use of resources. As John Donahoe, the CEO (Chief Executive Officer) of eBay Inc., mentioned, “the greenest product is the one that already exists, because it does not draw on new natural resources to produce” [17]. However, existing research has mainly focused on developing new products to meet the needs of long-life, maintainable, and expandable properties, but these approaches cannot extend the life cycle of existing products. It is difficult to make changes once resources, infrastructures, and activities have been committed to a certain product design [18]. Moreover, design for maintenance and repair or design for future expansion and modification can only be achieved with visible mature technologies [9]. In the case of rapid technology iteration, the method’s ability to extend the life cycle is becoming more and more limited. Therefore, under the current situation of a rapid spread of IoT (Internet of things) technology and new consumer needs constantly being stimulated, for a large number of products that are circulating or used in the market, how to extend their life cycle to reduce resource consumption and waste stream generation is a very urgent task, and this study aims to propose a new GED strategy to solve this problem and define a calculation method for evaluating the GED performance. The framework of the GED strategy and its evaluation is shown in Figure 3.

## 2. Green-Extension Design

### 2.1. The Definition of Green-Extension Design

**Figure 4:**
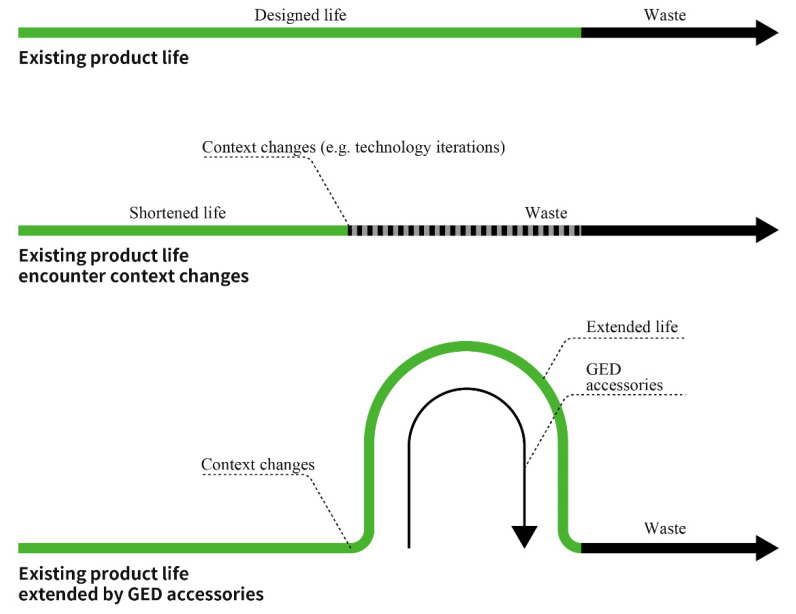
GED strategy for extending the existing product life.

Upgradability is defined as the ability of a product to continue being useful under changing conditions by improving the quality, value, and effectiveness or performance. This research proposes a new green design strategy, i.e., developing adaptable accessory products that can improve the performance or value of existing products that have been put into use or on the market, so as to give them the ability to continue working under a changed context, as illustrated in Figure 4. For example, in the context of the development of the IoT, more and more users have begun choosing smart locks. Smart locks are facing the problem of elimination despite an effective mechanical function. Through the development of a smart electronic accessory for rotating the thumb-turn of mechanical locks, old locks can be upgraded to smart locks without changing the original parts, which directly extends the life cycle of the original product and avoids the existing lock being discarded. Considering the literature discussion and research on green design, the definition of Green-Extension Design is as follows: Green-Extension Design is a design strategy that gives existing products that have been put into use or on the market the ability to continue to work under a changed context by developing adaptable accessories.

### 2.2. Initial GED Competitiveness Factors

GED is an easy-to-understand strategy that can quickly inspire designers to develop green accessories based on existing products. However, as a new green design strategy, it is necessary to evaluate performance and avoid blindly developing GED accessories. If discussed at all, ecoperformance was usually explored in terms of the tradeoffs involved with technological or economic performance [19]. Consequently, establishing an effective evaluation method for GED, guiding designers and companies to work in the right way, is the main purpose of this research. The challenge of responding appropriately to concerns about the natural environment has changed many aspects of the way businesses operate and has become an integral part of purchasing, marketing, and corporate strategy [20]. Whereas environmental responsiveness was once viewed as involving compliance, expense, and tradeoffs with other corporate goals, it is now increasingly being portrayed as an opportunity. Porter [21] proposed the “win–win” logic of being “green and competitive”. Products should enhance their competitiveness [22] since companies need to increase sales [23]. According to the literature review, competitiveness is important when evaluating green businesses. Thus, competitiveness was selected to evaluate the performance of GED. The concept of competitiveness of GED was defined as the evaluation criterion of the GED strategy. Since competitiveness is still a broad concept, competitiveness factors were introduced, defined as the important issues that affect competitiveness.

This research conducted a statistical analysis of user evaluations on e-commerce of selected representative GED samples and summarized the competitiveness factors that need to be referred to when developing GED products. In view of the wide scope of the term “product”, this study only discusses the competitiveness of electronic consumer products or areas that need to be upgraded to electronic consumer products. Online evaluation has an important impact on customers’ purchase behavior and enterprise product development [24]. Customers can browse Amazon’s reviews to help determine their purchase intentions. Companies can also use Amazon’s user reviews, collect user opinions, improve products, discover competitors’ information, and obtain market data. Accordingly, this research selected three representative samples with GED characteristics as cases, focusing on collecting and sorting out users’ reviews of products. It should be noted that, because the GED is a new design concept, there are not many representative products at present; thus, only three representative samples were selected: AUGUST smart lock (Manufacturer: August Home, San Francisco, CA, USA), SWITCHBOT switch robot (Manufacturer: Switchbot Global, Tokyo, Japan), and SWITCHBOT curtain robot (Manufacturer: Switchbot Global, Tokyo, Japan). The reasons for choosing these three products were as follows: (1) they are products for end consumers, not engineering components for professional engineers; (2) they are all installed outside of the original product, without the need to replace the original product; (3) they have been on the market for more than half a year and exhibit stable sales, thus qualifying their performance. The introduction of the three representative samples is shown in Table 1.

**Figure 5:**
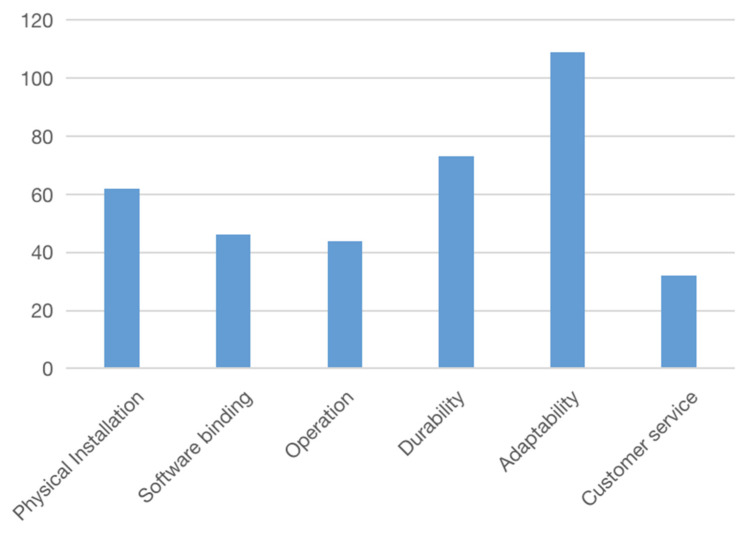
Competitiveness factors derived from GED samples.

For each representative sample, this study collected 100 copies of valid product-related reviews. Through sorting, six competitiveness factors were summarized, namely, physical installation, software binding, operation, durability, adaptability, and customer service, as shown in Table 2 and Figure 5.

According to the literature, price, advertising, brand, and quality have a significant impact on users’ purchasing intentions [25]. Considering that the brand factor is very complex, this study does not take this factor into discussion from the design point of view, i.e., it is assumed that the brand influence of different products is the same. In addition, the quality factor actually contains the durability, operation, and software relevance factors previously summarized in this research. Therefore, quality was not selected as a competitiveness factor. The research of Khan et al. [26] and Schoormans et al. [27] showed that product appearance has a significant impact on consumers’ purchasing behavior; thus, appearance was selected as a competitiveness factor. Lastly, this research preliminarily identified nine factors of GED competitiveness, namely, price, advertising, appearance, physical installation, software binding, operation, durability, adaptability, and customer service.

### 2.3. Competitiveness Evaluation and Analysis

In order to summarize the main components of GED competitiveness according to the nine factors, this research designed a user experiment. For the experiment, in order to make up for the lack of samples, this study virtualized six new GED representative samples according to the three on-sale samples’ and usage conditions; their specific description is shown in Table 3. The on-sale samples and virtual samples together constituted nine samples. In order to make the questionnaire easier to understand, the nine GED competitiveness factors were transformed into statements. For example, “price” was transformed into “the price of this product needs to be much lower than the price of buying a brand-new product”; this experiment used the method of an online questionnaire survey. Thirty participants were invited to join this experiment. Considering that the GED is an innovative design strategy, not all participants can understand and accept it quickly. Therefore, before selecting the final 30 participants, a selection questionnaire survey was carried out. In order to improve the effectiveness of the test, the population age distribution was limited from 18 to 55 years old, including 17 male participants and 13 female participants.

Using principal component analysis (PCA) to analyze the experimental results, a rotation component matrix was obtained, as shown in Table 4. In the PCA analysis, the commonly used varimax rotation method was selected. This method has the characteristics of a simple and clear structure. After rotation, each factor remained linear and uncorrelated. At the same time, the sum of the variance of each factor load was the largest, which improved the interpretation of the factors [28]. The evaluation items were sorted and grouped according to their importance. A factor load of 0.5 was used as the observation value, and the common influencing factors in each component were taken into consideration when naming the main components. Three main sets of competitiveness factors could be obtained.

When the three main components were extracted by principal component analysis, the cumulative total variance explained was 77.167%, indicating that the main components of these three dimensions were sufficient to represent about 77% of the GED competitiveness. The first principal component included three competitiveness factors: physical installation, software binding, and operation, which are mainly related to the usability of the product, reflecting that users are firstly concerned with whether the product is easy to use. It can be speculated that accessory products developed based on the GED strategy require DIY (Do it yourself) processing by users; hence, ease of use becomes particularly important. Usability refers to “the quality of interaction in terms of parameters, such as the time it takes to perform a task, the number of errors made, and the time to become a qualified user” [29]. The term “usability” also refers to ways to improve ease of use during the design process” [30]. Therefore, the first principal component (competitiveness) can be summarized as usability. The second principal component included three competitive factors: adaptability, customer service, and advertising. The It can be speculated that existing products vary in terms of shape, size, and performance; as such, it is important to consider if accessories can work with them. Consequently, the problem of adaptability can lead to after-sales related customer service consultation, whereas advertisements linked to the presale publicity and education of the product can avoid confusion related to adaptability to a certain extent. Therefore, the second principal component (competitiveness) can be summarized as adaptability. The third principal component included appearance, price, and durability, among which price was strongly negatively correlated. This result is in line with the expected speculation. As an accessory product, its price not only needs to be lower than that of new products, but it may also need to be much lower than that of new products to ensure its market competitiveness; otherwise, users will be more inclined to buy new products. As an accessory installed outside of the existing product, appearance will attract the attention of users. As a new concept product, it is understandable for users to request stability, whereas, for mature products, stability is a basic requirement and is not worth mentioning. Appearance and stability can represent the quality requirements of the product, and the negative price correlation can be understood as the demand for low prices. Therefore, the third principal component (competitiveness) can be summarized as cost-effectiveness. In summary, the GED competitiveness can be summarized into three main components: usability, adaptability and cost-effectiveness.

## 3. GED Competitiveness Model Construction

### 3.1. Weight Analysis of GED Competitiveness

The analytic hierarchy process was used to calculate the weight of the three competitiveness components. The analytic hierarchy process is a multi-objective decision-making method, which is used in the fields of economy, society, and management science. The main theoretical basis is to use the hierarchical structure to help decision makers have a deeper understanding of an uncertain situation and of research issues with multiple evaluation criteria, enabling solutions to complex decision-making problems [31,32]. Five experts in related fields were invited to conduct questionnaire evaluations. The final three competitiveness weights were the average of the evaluation results of five experts. The results are shown in Table 5. The results show that usability had the largest weight at 0.469, indicating that, for the GED, as an end-consumer-oriented design strategy which requires consumer DIY, usability is the most important. In other words, usability will determine whether the design can be accepted by consumers. Durability had a weight of 0.313, indicating that adaptability is an important decision-making factor. Adaptability will affect the space capacity of the product in the market. A higher adaptability can lead to greater market space. Cost-effectiveness had a weight of 0.218, indicating that cost-effectiveness is also an important decision-making factor. When the design meets the requirements of usability and adaptability, the cost control of the product also needs to be considered. Reducing costs and improving cost-effectiveness can further improve product market competitiveness.

### 3.2. Construction of the GED Competitiveness Model

Robert E. Lucas [33] proposed an endogenous growth model, which defines the production function including the contribution of human capital, as shown in Equation (1).

(1) $$Y=AK^{\beta }{\left(uhL\right)}^{1-\beta }h_{a}{}^{\psi }(\psi >0),$$

where *Y* is the total output, *K* is the stock of physical capital, *u* is the proportion of labor working-hours, *h* is the average quality of labor as measured by education level, *L* is the number of labors, *uhL* is defined as human capital, and $h_{a}{}^{\psi }$ reflects the overflow of human capital effect. *A* is a constant term, which represents the initial technical level.

On this basis, Wang et al. [34] proposed a production model based on human capital, as shown in Equation (2).

(2) $$lnY_{\left(t\right)}=C+a_{1}lnK_{\left(t\right)}+a_{2}lnH_{\left(t-3\right)}+a_{3}H_{a(t)}+R,$$

where *Y* is GDP, *K* is the stock of fixed capital, *H* is the stock of human capital or effective labor, defined as the total working-age population (excluding students in school) multiplied by their years of education, *H_a_* is the average number of years of education of workers, *C* is a constant term, *R* is a residual term, and *t* is the year.

The production function model provides a classic interdisciplinary analysis method of the relationship between each element and output. Through the transformation of the model, the impact of various input elements on output can be further analyzed. According to this model, the GED competitiveness can also be used as an output, which can be incorporated into the model setting framework of the production function to analyze the marginal contribution and influence mechanism of different competitiveness factors toward the overall GED competitiveness.

Therefore, this research further proposes the Green-Extension Design competitiveness model, as shown in Equation (3).

*G* = *w*1*U* + *w*2*A* + *w*3*C* + *Ri*, (3)

where *G* represents the total value of the GED competitiveness of a product, *U* represents usability, *A* represents adaptability, and *C* represents cost-effectiveness. *w*1, *w*2, and *w*3 represent the respective weights of the three competitiveness components. *Ri* is a constant and represents the initial competitiveness level of different product categories. When the *R* value of different categories is defined, cross-category GED competitiveness comparisons can be carried out.

### 3.3. Quantitative Calculation Analysis of GED Competitiveness

**Figure 6:**
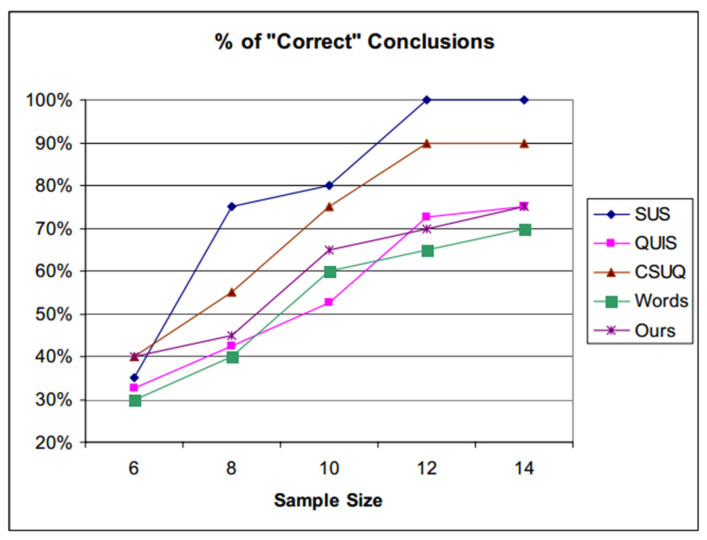
Correct conclusion comparison [40].

In the existing research, there are many kinds of evaluation methods for usability, and the most widely used are currently SUMI (Software Usability Measurement Inventory) [35], PSSUQ (Post-Study System Usability Questionnaire) [36], CSUQ (Computer System Questionnaire) [37], and SUS (System Usability Scale) [38]. Among them, the SUS scale is simplified with only 10 questions. The statements are concise and the questionnaire assessment is easy to implement. It can be used to evaluate the usability of physical products, such as electronic consumer products, and it can also be used for the interactive measurement of software interfaces, such as website pages and APP interfaces. A number of empirical studies have shown that SUS is more effective. Bangor [39] used a large number of sample experiments and found that the reliability coefficient of SUS is 0.91. Tullis et al. [40] showed that, when the sample quantity is limited, SUS can achieve the fastest effect, as shown in Figure 6.

**Figure 7:**
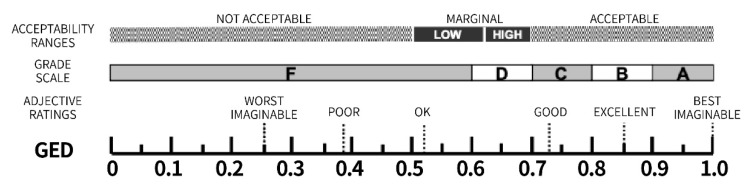
SUS scale of GED (based on [38]).

Therefore, this study used the SUS percentile scale as a reference and quantified it using a score of 0–1, which is convenient for unified dimensions with the other two competitiveness components, as shown in Figure 7.

Adaptability is an indicator that reflects the adaptation of the extended design to the original product, and the adaptability can be measured by testing the percentage success rate of adaptation, as shown in Equation (4). It is recommended to test no less than 30 objects.

*A* = *Sc*/*Tc*, (4)

where *Sc* represents successful cases, i.e., the number of samples successfully installed among the test samples, and *Tc* represents the total number of test samples. For example, to test the adaptability of a product, if 30 groups of users (*Tc* = 30) care selected, and the number of successfully installed groups is 25 (*Sc* = 25), then *A* = 0.833.

Cost-effectiveness is a relative concept, and it needs to be compared between competing products to reflect its meaning. Since the GED mainly competes with brand-new products that replace old products, the cost-effectiveness of GED can be measured by comparing the prices of brand-new products with similar functions, as shown in Equation (5).

*C* = *Np*/(*Gp* + *Np*), (5)

where *Np* represents the price of a brand-new product with similar functions, and *Gp* represents the price of a GED solution. The definition of the ratio is mainly to unify the dimension and follow a positive definition, whereby a larger value denotes better competitiveness. The price here includes the product price and installation cost. For example, product A needs to be updated now. The first option is to buy a brand-new replacement product at a price of 200 USD, and an installation fee of 100 USD is required; thus, *Np* = 300. At the same time, the other option is to buy an upgrade accessory for product A at only 50 USD, and there is no installation cost; thus, *Gp* = 50, *C* = 0.857.

As a constant item of different categories, *Ri* can be obtained only after a large number of tests on products of different categories. Since the current GED is a relatively new strategy, the number of representative samples that can be studied is not sufficient; therefore, the calculation of *Ri* is not discussed at this stage, and it will only be used as a part of the model for subsequent research.

## 4. Conclusions

This research firstly proposes a new strategy to extend the life of existing products facing technological elimination issues. By statistically analyzing the user reviews of representative samples on an e-commerce platform, the GED competitiveness and its factors were summarized. On this basis, this research further analyzed the weight of GED competitiveness components through expert evaluation and established a quantifiable calculation model of GED competitiveness.

In the trend of product upgrading brought about by the rapid development of the Internet of things, the GED aims to design and develop functional accessories that adapt to existing products, to help consumers easily and quickly upgrade and transform household facilities, to reduce the technical elimination and abandonment of products, and to ultimately reduce resource waste and environmental pressure.

At the industrial application level, enterprises can use the factors of GED competitiveness to assist in defining product development and enhancing product innovation and market competitiveness. Meanwhile, using the GED competitiveness model to evaluate a design project can reduce product development risks. Therefore, this research is of great significance for enterprises to establish a benign business model when developing green products. Only when the enterprise can develop green products with market competitiveness can green design develop continuously.

Future research can expand the research scope beyond consumer electronics, as well as optimize the parameters of the model, by expanding the number of tested samples when GED-related products grow large.

## Figures and Tables

**Table 1:** GED representative samples.

GED Samples	Description
Smart lock control accessories 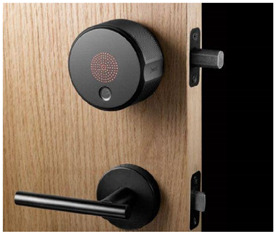	Simple installation: one only needs to replace the rear panel of the door lock, with no need to change the lock; Supports APP unlocking; Virtual keys can be distributed through APP for easy management.
Smart light control accessories 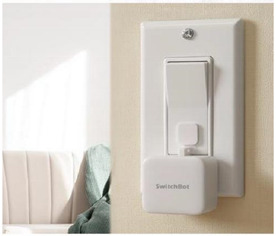	Simple installation: there is no need to replace the original switch; It can be adapted to many types of switches; Supports APP remote control.
Smart curtain control accessories 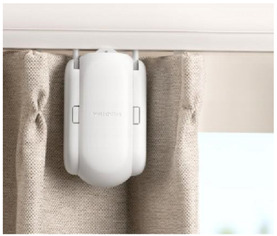	Installation is simple: there is no need to punch wires; Supports APP remote control; Supports intelligent scenes such as timed opening and closing of curtains.

**Table 2:** Online reviews derived from GED samples.

Competitiveness Factors	Description	Frequency
Physical installation	Manual is hard to understand	8
	Installation needs drilling holes	6
	Installation in a wrong position	25
	Do not know how to install	13
	Installed over 2 times	10
Software binding	Met error when binding	7
	Confused with the indicator light	8
	Cannot bind the device	7
	Complicated binding	14
	Cannot bind the device after deleting	9
Operation	The thumb turn does not feel good	5
	Not convenient when replacing batteries	8
	Not convenient when charging	11
	Hard to set the device	14
	APP is complicated to interact	7
Durability	Not durable	7
	Connection often breaks	20
	Some functions are not stable	10
	The adhesive is not strong enough	15
	Battery cover falls off	5
	Powers off in a short time	16
Adaptability	Lock size not compatible	12
	Lock panel not compatible	14
	Lock cylinder not compatible	18
	Switch panel not compatible	13
	Touch panel not compatible	6
	The curtain rod is too thick to fit	11
	Torque is insufficient	26
	Old-fashioned curtains not compatible	9
Customer service	Customer service response is too slow	11
	Problem is still not solved after consulting with customer service	14
	No response when consulting	7

**Table 3:** GED samples for testing.

Sample	Description
Smart control light accessory	A pressing mechanical arm that can be installed on the switch panel of the light; realizes the wireless intelligent control of the switch of the light, with no need to replacing the switch panel.
Smart control curtain accessory	A moving device that can be installed on the curtain rod to pull the curtain; easily realizes the wireless intelligent control of the curtain switch without replacing it.
Smart control lock accessory	A rotary knob device that can be installed on the lock panel to realize wireless intelligent control of the door lock without replacing it.
Smart control air conditioner accessory	A pressing mechanical arm that can be installed on an air-conditioner switch panel; realizes wireless intelligent control of the air-conditioner switch without replacing it.
Smart control water heater accessory	A pressing mechanical arm that can be installed on the switch panel of a water heater; realizes the functions of wireless intelligent control of the water heater switch and timed heating without replacing it.
Smart control window accessory	An automation device that can be installed on a window; realizes wireless intelligent control of the window without replacing it.
Smart intercom control accessory	A pressing mechanical arm that can be installed on an intercom; realizes wireless intelligent control of the intercom switch without replacing it.
Smart control socket accessory	A pressing mechanical arm that can be installed on a socket; realizes wireless intelligent control of the socket without replacing it.
Smart control audio accessory	A pressing mechanical arm that can be installed on an audio accessory; simply and quickly realizes wireless intelligent control of the audio accessory, with no need to replace it.

**Table 4:** Factor loadings of the evaluation items (varimax with Kaiser normalization); only the factor loadings > 0.5 are shown.

Factors	Factor loadings		
	1	2	3
Physical installation	0.881		
Software binding	0.863		
Operation	0.836		
Adaptability		0.832	
Customer service		0.798	
Durability		0.685	
Appearance			0.812
Price			−0.755

**Table 5:** GED competitiveness components weights.

Competitiveness component	Factors	Weights
Usability	Physical installation	0.469
	Software binding	
	Operation	
Durability	Adaptability	0.313
	Customer service	
	Durability	
Cost-effectiveness	Appearance	0.218
	Price	
	Advertisement	
